# Darwinism for the Genomic Age: Connecting Mutation to Diversification

**DOI:** 10.3389/fgene.2017.00012

**Published:** 2017-02-07

**Authors:** Xia Hua, Lindell Bromham

**Affiliations:** Centre for Macroevolution and Macroecology, Research School of Biology, Australian National University, CanberraACT, Australia

**Keywords:** molecular evolution, macroevolution, phylogeny, comparative studies, reproductive isolation

## Abstract

A growing body of evidence suggests that rates of diversification of biological lineages are correlated with differences in genome-wide mutation rate. Given that most research into differential patterns of diversification rate have focused on species traits or ecological parameters, a connection to the biochemical processes of genome change is an unexpected observation. While the empirical evidence for a significant association between mutation rate and diversification rate is mounting, there has been less effort in explaining the factors that mediate this connection between genetic change and species richness. Here we draw together empirical studies and theoretical concepts that may help to build links in the explanatory chain that connects mutation to diversification. First we consider the way that mutation rates vary between species. We then explore how differences in mutation rates have flow-through effects to the rate at which populations acquire substitutions, which in turn influences the speed at which populations become reproductively isolated from each other due to the acquisition of genomic incompatibilities. Since diversification rate is commonly measured from phylogenetic analyses, we propose a conceptual approach for relating events of reproductive isolation to bifurcations on molecular phylogenies. As we examine each of these relationships, we consider theoretical models that might shine a light on the observed association between rate of molecular evolution and diversification rate, and critically evaluate the empirical evidence for these links, focusing on phylogenetic comparative studies. Finally, we ask whether we are getting closer to a real understanding of the way that the processes of molecular evolution connect to the observable patterns of diversification.

## Introduction

Darwinism unites genetics (heritable characteristics) with population biology (change in frequency of heritable traits through differential reproduction) and biodiversity (formation of new lineages through speciation). The neo-Darwinian synthesis codifies the belief that these processes are all parts of a single evolutionary process, such that the generation of genetic variation that makes individuals differ from each other feeds the change in the characteristics of populations by selection and drift which drives speciation and the diversification of lineages. Thus the neo-Darwinian synthesis “puts an equals sign between microevolution and macroevolution” (Dobzhanksy, 1937). Most biologists accept this unified view of evolutionary process. Yet evolution is still predominantly studied in a remarkably partite fashion, with most researchers concentrating on one particular aspect of this process. While discipline specialization is a necessary part of modern science, there are some evolutionary phenomena that require an appreciation of many different levels of biological organization.

One such phenomenon is the observed correlation between differences in the mutation rate, estimated by comparing DNA sequences of different species, with net diversification rate, estimated from the number of extant species in different lineages (Barraclough and Savolainen, 2001; Pagel et al., 2006; Eo and DeWoody, 2010; Lanfear et al., 2010; Duchene and Bromham, 2013; Bromham et al., 2015). This relationship might initially seem surprising: how could the rate at which mistakes or damage are repaired in individual genomes lead to differences in species richness between lineages? The correlates of diversity are usually examined at the level of lineages, regions and biological communities. For example, diversification rate has been found to be associated with life history, dispersal rate, generalized feeding strategies, geographic range size, environmental energy and latitude (Cardillo, 1999; Cardillo et al., 2003; Davies et al., 2004; Phillimore et al., 2006). But evolutionary change must ultimately start with heritable changes to genetic information, so the supply of variation is a critical first step in the formation of new evolutionary lineages.

Our aim with this Hypothesis and Theory paper is to examine the possible links in the chain of causation that connects the average mutation rate, estimated by comparing nucleotide sequences, to differences in lineage net diversification rate, as measured from molecular phylogenies. In order to investigate how mutation rate could be linked to diversification rate, we need to consider how an increase in the supply of genetic variation could speed the rate of formation of new species. To do this we will first consider why mutation rate varies between species. Then we will examine the factors that shape the number and type of mutations that become fixed features of the genome within a population (substitutions). Then we consider how the accumulation of these substitutions can cause population to diverge from each other, until the genetic differences between their genomes prevent populations from exchanging genetic material. At this point, the populations can no longer interbreed, and we consider them separate species. Lastly, we propose a conceptual approach to relating the formation of new species to bifurcations on molecular phylogenies.

We explore both the theoretical foundations and empirical evidence for the links in the causal chain between mutation rates and macroevolution. These connections bring together topics typically considered in different biological disciplines, namely molecular evolution, population genetics and macroevolution, but we illustrate some of the approaches that allow us to effectively view all of these processes simultaneously by comparing DNA sequences between individuals, populations, species and lineages. Because the relationship has been noted by comparing gene sequences between lineages, we confine our discussion to mutations that change nucleotide sequences. However, there are many other kinds of genetic change that may also be important drivers of diversification, such as chromosomal rearrangement, regulatory changes or epigenetic modification.

## Mutation Rates Evolve

To explain why variation in mutation rate is associated with differences in diversification rate, the first topic we have to address is why species differ in their average mutation rate. Mutation rate is often in the background of molecular evolutionary studies. It is frequently assigned an arbitrary constant value, providing a steady drip of genetic variation into populations. But mutation rate is a dynamic and highly variable phenomenon, changing across the genome and over time, varying among individuals and lineages. We should not be surprised that mutation rate is highly responsive to evolutionary pressures: it is, after all, the fundamental process at the heart of all evolutionary change.

Because we wish to examine the links between diversification rate and the rate of molecular evolution as measured from DNA sequence comparisons, we will consider only point mutations that change single nucleotides in gene sequences. Point mutations arise when damage to DNA is imperfectly repaired or errors in DNA replication are not fully corrected, resulting in permanent change to the base sequence such that the changed sequence will be included in any subsequent copies. DNA is a large, complex molecule: it is inevitable that it will occasionally suffer damage that affects the genetic information it encodes. In addition, the genome must be replicated every time a cell divides, so even a phenomenally low replication error rate (typically only one error per billion bases copied) will result in frequent changes to the DNA sequence. To protect the integrity of the genetic information needed to make essential components of the organism, there is a diverse and sophisticated set of cellular machinery that is directed at detecting and correcting any damage to the DNA or mistakes made in copying the genome. The costs of DNA repair to the cell are not well known, but it seems fair to say that investment in repair apparatus must come at cost of other cellular functions. Therefore, while we expect organisms to invest resources in reducing the risk of harmful mutation, there might come a point where the payoffs of further reduction in mutation rate are outweighed by the increasing costs of DNA fidelity. All species must walk a tightrope, finding a balance between fidelity and error, but they may find different points of balance between repair and mutation (Denamur and Matic, 2006; Bromham, 2009; Lynch, 2010).

One aspect of this balancing act, initially proposed on the basis of information theory, is the need to maintain mutation rates below an “error threshold,” the copy error rate above which a replicating sequence effectively goes extinct by failing to produce sufficiently faithful copies of itself (Eigen, 1971). In the earliest life forms, this may have led to a trade-off between genome size and mutation rate: for a given per-base error rate, a small genome is more likely to be copied without error, but a small genome may also have limited capacity for evolving mechanisms that reduce the mutation rate (Maynard Smith and Szathmáry, 1995). The error threshold model appears to place real-world constraints on the evolution of RNA viruses (Holmes, 2003). The reliance of RNA viruses on the error-prone reverse transcriptase to complete their life cycle endows them with a mutation rate more than an order of magnitude greater than DNA viruses (Sanjuán et al., 2010). While the high mutation rate in RNA viruses has been considered to contribute to their rapid evolvability, it may also place restrictions on adaptation by limiting genome size. Small genomes may speed replication, but it could also place constraints on coding capacity. Therefore it has been suggested that the high mutation rate of RNA viruses may, by limiting genome size, constrain the capacity for evolving new genetic features (Holmes, 2003). Increase in mutation rate beyond the error threshold has been reported as causing extinction of yeast lines (Herr et al., 2011a).

It has been suggested that many species settle on a similar point of balance between the per-genome copy error rate and genome size that represents a chance of error roughly once in every three hundred genome copies (Drake et al., 1998), at least relative to the “non-frivolous fraction” of the genome in which mutations can influence fitness (Drake, 1991). A larger genome with more functional sequences provides more targets for deleterious mutations, so a lower per-base mutation rate is required to have the same per-genome-replication risk of fitness-damaging mutations. The balancing point for the per-genome mutation rates has been most frequently examined for single-celled organisms. It is less clear whether the same relationship between genome size and mutation rate applies to large, complex multicellular organisms. There is surprisingly sparse data on the association between mutation rate and genome size in large multicellular organisms (Bromham et al., 2015), but observed patterns have been considered consistent with an overarching relationship between the effective genome size and the per-generation mutation rate (Lynch, 2010).

The balancing point between the costs and benefits of mutation and repair can be altered by circumstance. Both theory and experiment have suggested that populations of microbes subject to rapidly changing conditions can show a transient increase in mutation rate because “mutator” alleles that increase the rate of mutation can hitchhike to fixation through their association with advantageous mutations (Chao and Cox, 1983; Haraguchi and Sasaki, 1996; Sniegowski et al., 1997; Taddei, 1997; Giraud, 2001). These theoretical models and laboratory experiments have found additional support in observations from medical settings. For example, bacteria infecting the lungs of patients with cystic fibrosis can only persist if they can adapt to ongoing antibiotic treatment and a changing environment as the patient’s lung condition deteriorates, resulting in a higher frequency of bacteria with raised mutation rates (Oliver et al., 2000; Oliver and Mena, 2010).

Both models and experiments suggest that it is possible for mutation rates to be shaped by adaptation to circumstance. However, most of these experiments and simulations produce only transient increases in mutation rate, as the association between the mutator allele and beneficial mutations may be uncoupled by recombination, and the burden of deleterious mutations will result in selection against the mutator allele (McDonald et al., 2012). Can selection produce long-term differences in mutation rate between species through balancing the costs and benefits of mutation and repair? There are several observations that suggest it can. Firstly, in many populations there is naturally occurring heritable variation in mutation rates. For example, individuals can vary in the proofreading efficiency of their polymerase enzymes or their mismatch repair systems (Oliver et al., 2002; Sundin and Weigand, 2007; Reha-Krantz, 2010). Mutations that disable essential DNA repair genes can lead to disease phenotypes with dramatically increased mortality for affected individuals (Bradford et al., 2011), but slight variations in DNA repair genes that cause relatively mild reductions in DNA repair efficiency and fidelity can also influence fitness (e.g., Mohrenweiser et al., 2003; Gokkusu et al., 2013; Kabzinski et al., 2016). Although most studied “antimutator” alleles are changes that can compensate for deficiencies in mutator strains (Herr et al., 2011b), there is some evidence of naturally occurring alleles that can increase DNA repair efficiency or copy fidelity (Schaaper, 1998; Foury and Szczepanowska, 2011). The existence of spatial variation in DNA repair enzymes provides further evidence of the evolvability of mutation rate in natural populations (Miner et al., 2015; Svetec et al., 2016). So the raw material for selection to act on mutation rates does not seem to be lacking in natural populations.

Secondly, it seems fair to suppose that DNA repair is costly, such that organisms must find a level of investment that maximizes survival yet minimizes costs. Higher DNA repair efficiency may come at cost of other key cellular functions. For example, costs of repair may explain why the induction of resource-intensive stress response pathways in bacteria can lead to an increased mutation rate (Torres-Barceló et al., 2013). The potential trade-off between investment in DNA repair and other cellular functions is supported by the observation that some DNA repair systems are inducible under mutagenic conditions. For example, plants can have repair systems that are turned on or increased under high UV conditions, to prevent an upsurge in DNA damage (Ries et al., 2000). This suggests that maximum DNA repair efficiency is not maintained at all times, instead the repair effort is scaled to a level that allows the maintenance of cellular processes and reproductive potential. The metabolic cost of DNA repair is supported by observations that individuals in poor condition or subjected to mild stress can have elevated mutation rates, presumably because they are unable to invest as much in DNA repair (Agrawal and Wang, 2008; Goho and Bell, 2000). For example, it has been reported that male birds with lower levels of antioxidant carotenoids have higher rates of DNA damage and also reduced survivorship and lower mating success (Freeman-Gallant et al., 2011).

Thirdly, species can adapt to different levels of risk of mutation through altered investment in DNA repair. Species living in highly mutagenic environments might require greater investment in DNA repair in order to be able to maintain a persistent population against mutational meltdown. Microbes living in high-altitude lakes, exposed to high salinity, high levels of UV radiation and high concentrations of heavy metals, have been shown to have enhanced levels of DNA repair (Albarracin et al., 2012). Bacteria adapted to survive long periods of desiccation, which results in accumulation of DNA double-strand breaks, can have enhanced DNA repair that incidentally allows them to survive other mutagens such as ionizing radiation (Mattimore and Battista, 1996). *Escherichia coli* selected to survive ionizing radiation can evolve DNA repair proteins that are more efficient and less prone to inhibition by perturbations of normal metabolism, potentially making these repair proteins able to function under a broader range of environmental conditions (Piechura et al., 2015).

Conversely, species living in low mutagen environments may need to invest less in DNA repair. For example, species living in low UV environments may have lost some of their photolyase repair genes (Lucas-Lledó and Lynch, 2009). Populations can have consistently different levels of efficiency of UV-induced DNA repair, apparently reflecting different points of adaptation of DNA repair efficiency to match local environmental conditions. For example, water fleas from natural ponds with higher levels of UV exposure have more efficient DNA repair of light-induced mutation (Miner et al., 2015) and fruit fly populations from different latitudes show different levels of UV repair efficiency (Svetec et al., 2016). The adjustment of DNA repair to the level of mutation risk might also explain the puzzling lack of evidence for the prediction that basal metabolic rate should have a direct influence on the mutation rate, due to the production of free oxygen radicals that can damage DNA (Gillooly et al., 2007). Although this relationship has been modeled based on body size and temperature, comparative studies have found no significant variation of rate of molecular evolution with metabolic rate, above and beyond the association with other life history traits such as body size or generation time (Bromham et al., 1996; Lanfear et al., 2007; Galtier et al., 2009b). One possible explanation for the lack of a significant association between metabolic rate and mutation rate is that DNA repair efficiency may be adjusted to ameliorate any additional damage, so that species with high metabolic rates also evolve greater levels of protection against damage from free-oxygen radicals (Lanfear et al., 2007).

DNA repair proteins thus can be considered as a “highly adaptable scaffold readily tailored by evolution to the requirements for genome maintenance in each particular organism” (Piechura et al., 2015). Evolution shapes mutation rates just as it shapes other species traits, balancing costs and benefits to suit the species form and lifestyle. In the next section, we will consider how differences in mutation rates between species can vary predictably with species traits.

## Species Traits Influence Mutation Rate Evolution

Given that DNA repair is likely to impose a non-trivial cost on individuals, that higher rates of mutation can lead to lower survival and reproduction, and that individuals show genetic variation in DNA repair efficiency, we should expect selection to find a balance between the cost of repair and the cost of mutation. But the balance between these competing costs might vary with other species characteristics (Sniegowski et al., 2000).

In multicellular organisms, the costs of mutation are expected to increase with increasing body size (Bromham, 2011). The larger the body, the more cell generations it takes to build it. Every cell division requires the genome to be copied in its entirety, and every genome replication brings the risk of copy errors that might ruin important DNA sequences. The influence of number of genome replications on the mutation rate is supported by the observation of higher per-generation mutation rates in males than in females. Due to the large number of cell generations required for sperm production, the male germline is copied many times more per generation that the female germline, and so the majority of *de novo* mutations arise in males (Wilson Sayres and Makova, 2011). Furthermore, germline mutations accumulate with male age, suggesting that the increasing number of germline divisions per gamete results in more mutations (Kong et al., 2012; Venn et al., 2014). Species with stronger potential for sperm competition, and therefore selective pressure to increase sperm production, have been found in some cases to have higher rates of molecular evolution (Bartosch-Harlid et al., 2003), though this pattern is not supported in all studies (Wilson Sayres et al., 2011). The influence of number of DNA replications on mutation rate has been proposed as the explanation for the generation time effect on rates of molecular evolution, on the assumption that species with faster generation turnovers copy their germline DNA more often per unit time (Gaut et al., 1992; Bromham et al., 1996; Welch et al., 2008; Thomas et al., 2010; Lehtonen and Lanfear, 2014).

However, the copy error effect on its own does not seem sufficient to explain the observed life history patterns in mutation rate (Nabholz et al., 2008; Welch et al., 2008; Gaut et al., 2011; Hua et al., 2015). The difference in mutation rates between species is not commensurate with the difference in number of genome copies per unit time. For example, mice can go through 50 generations for every human generation, yet the rate of molecular evolution in mice is only a few times faster than that in humans. This suggests that the influence of copy number on mutation rate is modulated by other factors. Other life history characteristics have a significant relationship with mutation rate above and beyond their covariation with generation time, particularly fecundity and longevity (Nabholz et al., 2008; Welch et al., 2008). The significant association between generation time and molecular evolution rates may be partly due to it covarying with other causal traits, but being measured more reliably. Most studies of longevity are based on maximum recorded lifespan, which is strongly influenced by number of observations made (that is, it can only go up as more data points are included), so it is possible that for species with relatively little data on longevity, generation time is a better proxy for life history differences.

What effect will selection for longer life spans have on the evolution of a species’ mutation rate? Larger-bodied species tend to have longer lives, requiring the maintenance of more genome copies from incidental damage over a longer time period. Unrepaired damage to any cell’s genome can result in life-shortening damage, such as somatic mutations causing cancer. In particular, the accumulation of mutations in mitochondrial genomes has been proposed as a driver of aging (Fridovich, 2004; Kujoth et al., 2005; Loeb et al., 2005; Larsson, 2010; Yang et al., 2013). Reactive oxygen species produced in the mitochondria as a byproduct of metabolism can cause damage to DNA and other biomolecules. As damage accumulates over time, mitochondrial function may be impeded. So evolving a longer life span may require increased investment in cellular mechanisms that reduce the overall mutation rate in order to keep lifetime mutation risk to a tolerable level. For example, the mutation rate in long-lived species might be reduced by greater investment in mechanisms that prevent oxidative damage (Pamplona and Barja, 2011). This prediction is borne out by observations that long-lived species of mammals, birds and fish tend to have lower per-base mutation rates (as measured by the synonymous substitution rate), above and beyond the association between longevity and other aspects of life history (Nabholz et al., 2008; Welch et al., 2008; Galtier et al., 2009a,b; Hua et al., 2015).

Yet, molecular phylogenetic studies measure the germline mutation rate, not the somatic mutation rate. Although there must be evolutionary pressure on the mutation rate in both somatic and germline cells, the somatic mutation rate need not be the same as the mutation rate in the germline. Indeed, one of the possible evolutionary advantages of multicellularity is the ability to set aside a quiescent germ line in which DNA (particularly organelle DNA) is relatively protected from the harmful byproducts of metabolism, and for which expression levels are kept at a minimum (Bendich, 2010). Keeping germline copies in a quiescent state might be particularly valuable for mitochondrial genomes, hence the evolution of separate sexes: the mitochondrial genomes of motile and metabolically active male gametes can be discarded at fertilization, leaving only the quiescent, relatively unimpaired mitochondria from the immobile female gamete (Allen, 1995; de Paula et al., 2013).

While there is good reason to believe that selection acts on mutation rates, the power of natural selection to shape DNA repair might in itself by limited by species traits, specifically by traits that influence the mutation rate (affecting the supply of variation) and population size (affecting the power of selection). In small populations, fewer mutations that improve DNA repair will arise (Baer et al., 2007), and selection will be less effective at promoting slight improvements to DNA repair or removing mutations that increase the mutation rate, potentially limiting the effectiveness of selection in optimizing mutation rate (Knight et al., 2005; Lynch, 2007; Hodgkinson and Eyre-Walker, 2011).

Whatever the mechanistic or evolutionary forces that shape the differences in mutation rate between species – whether an incidental effect of copy frequency, an adaptive compromise between competing costs, or some other phenomena – the practical upshot is that even closely related species can differ in their average rate of mutation. So the rate of supply of new genetic variation to a species’ gene pool is at least partly dependent on a variety of species traits that influence the mutation rate. What effect will differences in the supply of variation have on the evolutionary process? The role of mutation rate in governing the rate of evolution has been given relatively little attention for non-microbial taxa, perhaps due to a general conviction that adaptation in complex multicellular creatures is typically not mutation-limited. But we should not be quick to dismiss the rate of mutation as playing an important role in population diversification. Although there is debate about the relationship between mutation rate, standing variation, and rate of adaptation (Barrett and Schluter, 2008), empirical studies suggest that adaptation to rapid environmental change or strong selection pressures can come from both standing variation (existing alleles) and *de novo* mutations (Hartley et al., 2006; Durand et al., 2010; Jerome et al., 2011). Even if adaptation is not mutation limited, population divergence can also be driven by non-adaptive genome evolution that may be influenced by the mutation rate.

Species-specific differences in mutation rate will only influence the evolutionary rate of lineage divergence if the supply of variation influences the rate of fixation of genetic differences: that is, if the mutation rate at least partly determines the substitution rate. So now that we have explored the evolutionary factors that shape the supply of variation to populations at any point in time, we need to consider how the supply of variation through mutation contributes to rates of genome evolution.

### Mutation Rate Influences Substitution Rate

Mutations occur in individual DNA molecules. For a mutation to be detected as a consistent difference in the DNA sequences sampled from different populations or species, it must clear several hurdles. First it must be copied from the genome it occurred in and passed on to that individual’s offspring, in order to enter a new generation. If the mutation is included in one or more offspring in the next generation, then each of those individuals has a chance to reproduce and pass the mutation to their offspring. While the mutation is at low frequency in the population, there is a high chance of being lost by chance if those few individuals fail to reproduce. If it increases in representation in the population, the mutation becomes established as a polymorphism, carried by some but not all members of the population. Eventually it may rise in frequency until it replaces all other variants in the population, so now all new individuals born in that population will carry a copy of that mutation (barring a new mutation occurring at the same site). At this point, we call the mutation a substitution and say that it has been fixed in the population. In this section, we will consider the factors that influence the rate at which new mutations become substitutions, thereby contribute to population divergence.

For each neutral mutation that has no effect on fitness, such as most synonymous substitutions that change the DNA sequence of a gene but not the amino acid sequence it codes for, the probability of being passed to future generations is entirely due to chance, and so its frequency in the population is determined only by the mutation rate and the effective population size (*N_e_*). However, the observed level of standing genetic variation within populations is often much lower than would be expected (Lande, 1976; Turelli, 1984; Lynch and Hill, 1986) if the amount of variation was shaped only by the processes of mutation and genetic drift (Frankham, 2012; Hodgins-Davis et al., 2015). This suggests that that there must be additional factors limiting the accumulation of genetic variation in populations (Eyre-Walker and Keightley, 2007).

For non-neutral mutations, such as most non-synonymous mutations that change the amino acid sequence of a protein, their frequency in the population will be determined not only by the mutation rate and the effective population size, but also the selection coefficient of the mutation (*s*). The influence of chance events on allele frequencies is potentially much greater in small populations. For example, the lucky survival and reproduction of an individual with a mutation that slightly lowers fitness will have a proportionally greater effect on allele frequencies in a small population than in a large population. In a large population, random fluctuations in allele frequencies are less likely to result in chance fixation, because they have proportionally less effect on average frequencies from one generation to the next. The larger the population, the less influence of chance events on allele frequencies. Therefore a beneficial mutation (*s* > 0) is more likely to increase in frequency in larger population than in a smaller population. Mutations with a substantial fitness costs (*s* << 0) will not become substitutions, as they will be removed from the population by purifying selection. But slightly deleterious changes (*s* < 0) will be nearly neutral and are more likely to go to fixation by chance in smaller population (Ohta, 1992).

We can describe the probability that a new mutation will becomes a substitution, found in all genomes in the population. We call this probability the fixation probability P_fix_. Theoretical studies provide an analytical approximation of the fixation probability (Waxman, 2011):

$$P_{fix}=\frac{1-E[e^{-4N_{e}sX(T)}]}{1-e^{-4N_{e}s}}.$$

This approximation accounts for a general situation where the values of effective population size and selection coefficient can change over *T* amount of time. So *T* = 0 means the population has constant effective population size and selection coefficient. According to the equation, the fixation probability depends on the product of the effective population size and the selection coefficient of the mutation, and on the frequency of the mutation at time *T* [*X*(*T*)].

**FIGURE 1:**
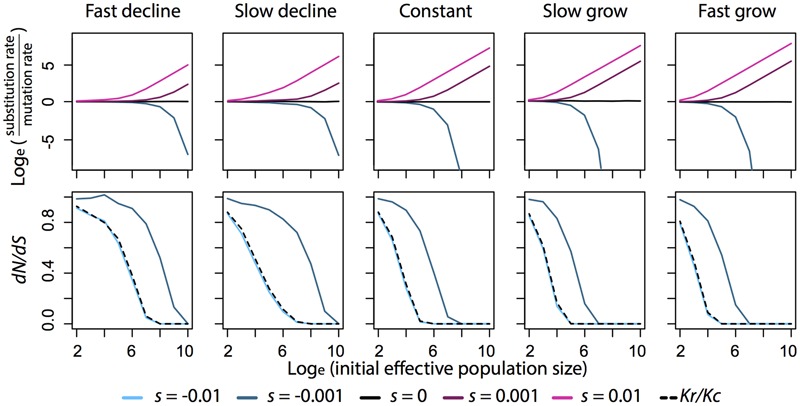
**Relationship between substitution rate, *dN/dS, Kr/Kc*, and effective population size (*N_𝜀_*) under different scenarios of population growth and selection coefficient (*s*).** Fixation probability is calculated by the general formula described in the text. To approximate the expectation value in the formula, 500000 replicates of an allele are simulated under a Wright-Fisher model described in Waxman (2011). In each simulation, selection coefficient stays constant and population size changes deterministically over generations. When population grows, the final population size is 1.9 times the initial population size. When population declines, the final population size is 0.1 times the initial population size. Fast population change takes 20 generations to reach the final population size. Slow population change takes 200 generations. Substitution rate is then calculated from the fixation probability and is plotted on natural log scale with respect to mutation rate, so when substitution rate equals mutation rate, the value is 0 in the plots. *dN/dS* is calculated as the ratio between the fixation probability of a slightly deleterious mutation (*s* = -0.001) to that of a neutral mutation (*s* = 0). *Kr/Kc* is calculated as the ratio between the fixation probability of a deleterious mutation (*s =* -0.01) to that of a slightly deleterious mutation (*s* = -0.001).

Over evolutionary time periods, the overall substitution rate in the population approximates the product of the mutation rate in the population and the fixation probability of each mutation, that is, 2N_e_uP_fix_, where *u* is the mutation rate. When most mutations are neutral (*s* = 0) and when effective population size stays constant, P_fix_ depends only on the initial frequency of a mutation. For a new mutation occurring in a population of diploid individuals, its initial frequency is 1/2N, where *N* is the number of individuals in the population. Under these conditions, substitution rate equals *uN_e_/N*. To investigate the impact of changing effective population size over time on the substitution rate, we simulated changes in the frequency of a mutation when the effective population size increases, decreases, or stays constant. The resulting frequencies were used to calculate the expectation value of e^-4N_e_sX(T)^ in the equation for P_fix_. Results suggest that changing the effective population size does not have a significant effect on the neutral substitution rate (**Figure 1**). Therefore, the rate of neutral substitutions always approximates *uN_e_/N*, regardless of the demographic history of the population.

Theory predicts the rate of fixation of neutral mutations will be unaffected by population size. The substitution of beneficial mutations will be faster in large population and the effect is greater in an expanding population (**Figure 1**). The substitution of deleterious mutations will be faster in small populations and the effect is greater in a declining population (**Figure 1**). Given that advantageous mutations are relatively rare compared to deleterious mutations (Eyre-Walker and Keightley, 2007), we expect smaller populations to have the greater overall substitution rate for a given mutation rate.

We can test these predictions by comparing different classes of substitutions in DNA sequence comparisons. Since all types of substitution rates are related to mutation rate, taking the ratio between any two of them will cancel out the influence of mutation rate. Therefore, a common test for the effect of population size on substitution rate is to correlate population size to the ratio between non-synonymous to synonymous substitution rates (*dN/dS*). Similarly, radical substitutions (from one class of amino acid residue to another) are more likely to be deleterious than conservative substitutions (within the same functional category) because radical substitutions cause more changes in the physiochemical properties of the protien, so the ratio between them (*Kr/Kc*) is expected to show the same pattern with population size as *dN/dS* (**Figure 1**; Zhang, 2000; Smith, 2003).

The predicted negative relationship between *dN/dS* and population size has been supported by observed correlations between *dN/dS* and life history traits that scale with population size (Nikolaev et al., 2007; Popadin et al., 2007; Lartillot and Delsuc, 2012; Romiguier et al., 2013; Figuet et al., 2014). It has also been supported by comparing sister lineages that differ in effective population size, such as domesticated populations to wild populations (Björnerfeldt et al., 2006; Wang et al., 2011) and island lineages to their mainland close relatives (Johnson and Seger, 2001; Woolfit and Bromham, 2005), although island lineages do not necessarily have smaller effective population size than mainland lineages (Wright et al., 2009; James et al., 2016). A puzzling exception is that *dN/dS* in birds shows no correlation to body size (which is often related to population size), but the correlation between *Kr/Kc* and body size is as predicted (Nabholz et al., 2013; Weber et al., 2014; Figuet et al., 2016). While there are many possible causes of the lack of relationship in birds, such as a lack of correlation between life history traits and historical population size or unreliable estimates of *dN/dS* (Lartillot, 2013), there is thusfar a puzzling lack of evidence that they provide an explanation for the lack of correlation between dN/dS and body size in birds (Figuet et al., 2016).

Interestingly, the formula of fixation probability never suggests a linear relationship between *dN/dS* and population size. In fact, we show in **Figure 1** that in the range of effective population size where it has strong negative relationship with *Kr/Kc, dN/dS* may show weak or even no dependence on population size particularly when population declines. So, a simple correlation test between *dN/dS* and population size may not be a robust way to test our theoretical understanding of substitutions against empirical data. We suggest that the formula of fixation probability should be explicitly accounted in future analyses on the relationship between population size and substitution rates. In particular, the formula can be written into a form similar to logistic regression, where the selection coefficient is the regression coefficient and the expectation term becomes a parameter in the link function to estimate.

We have considered the way that increases in mutation rate should flow through to increases in substitution rate, in many classes of substitution (neutral, advantageous, slightly deleterious). Now we can look at the way the accumulation of substitutions makes populations genetically distinct from each other. In the next section, we will consider how isolation between populations is achieved either by spatial separation or through the acquisition of genetic traits that reduce the chances of interbreeding. Once populations are isolated, they will accumulate unique sets of substitutions.

## Isolated Populations Acquire Different Substitutions

The simplest way for populations to become isolated is through the imposition of a physical barrier to the movement of alleles from one population to another. When a once-continuous population is divided by unsuitable habitat, there are three possible outcomes. One is that the barrier continues to prevent gene flow between the divided populations. The second is that the barrier is removed, allowing individuals or gametes to move between populations once more. The third is that one or both of the daughter populations acquires adaptations that allow them to overcome the barrier and re-establish contact with the other population. The first two processes are entirely determined by the frequency at which isolating mechanisms are created or removed. But the third possibility is a biological process and its frequency will depend not only on the nature of the isolating mechanism but also on the ability of either daughter population to adapt to the novel intervening habitat and overcome the barrier. Adaptation is found to occur on both new mutations and standing genetic variation (Olson-Manning et al., 2012). Standing genetic variation is also positively correlated with direct estimates of mutation rate (Vigouroux et al., 2002; Phillips et al., 2009). So, higher mutation rate could facilitate local adaptation, potentially allowing secondary contact between divided populations (Barton, 2001; Sexton et al., 2009).

It’s possible for substitutions to cause genetic isolation between populations in the absence of a physical barrier to interbreeding. Divergent selection for different substitutions within the population may contribute to non-random mating (Servedio et al., 2011). For example, divergent selection on flowering time has been detected in two genetically distinct populations of *Mimulus guttatus*, causing temporal isolation between the two populations (Lowry et al., 2008), and divergent selection on color pattern mimicry has been found to cause assortative mating in sister species *Heliconius melpomene* (Jiggins et al., 2001). Such substitutions can happen rapidly under local adaptation, generating genetically isolating populations (Servedio et al., 2011), so the effectiveness of this process will depend on how common these kinds of disruptive traits are, and on the rate of generation of relevant alleles by mutation. Therefore, the major limit on the evolution of non-random mating is the initial frequency of alleles associated with these disruptive traits in the populations, which is related to the mutation rate and the rarity of the traits. In theory, any substitutions that make individuals more likely to mate with individuals of the same population will facilitate genetic isolation between populations. The most general case is when substitutions make immigrants much less viable and/or fertile than local individuals (Servedio et al., 2011).

If divergent selection is removed, then non-random mating may breakdown. For example, light gradient has been found to drive divergent evolution of female sensory bias in some cichlid fish, such that females at different water depths prefer different male colors (Seehausen et al., 2008). But the same species living in turbid water does not show the same preference, presumably because light of the certain wavelengths is absorbed so fast that most water depths have monochromatic light, under which females cannot discriminate color (Seehausen and van Alphen, 1998).

We have now considered how sub-populations can acquire different sets of substitutions, either because physical barriers prevent movement of substitutions between them, or because divergent selection fixes different traits in different parts of a species’ range which directly or indirectly engender genetic isolation between populations. Now we will consider how the acquisition of these different sets of substitutions can contribute to genetic incompatibility between populations, paving the way for the formation of new species.

## Substitutions Lead to Incompatibility

Given some level of population isolation, genetic differences between populations will inevitably accumulate over time. If these genetic differences reduce the chance of successful reproduction between members of different populations, they contribute to the reproductive isolation (RI), preventing or reducing gene flow between populations. Broadly speaking, there are three ways that substitutions can contribute to reproductive isolation: by local adaptation of populations making hybrids unfit in either of the parent environment (e.g., Via et al., 2000), by influencing the chance of successful mating or fertilization (pre-zygotic isolation: e.g., Quinn et al., 2000), or by generating sets of alleles that reduce hybrid viability and/or fertility when mixed with alleles from other populations (post-zygotic isolation, or genomic incompatibility: e.g., Yamamoto et al., 2010).

However, there may be no clear line between the different ways that alleles contribute to RI. For example, local adaptation that involves changes to metabolic genes might involve co-ordinated changes to both mitochondrial and nuclear genes, potentially causing cytonuclear conflicts if mitochondrial alleles from one population are combined with nuclear alleles from another population (Johnson, 2010). Furthermore, if mutations conferring local adaptation are physically linked on the same chromosome to mutations causing genomic incompatibility, locally incompatible regions of the genomes (“speciation islands”) can reduce interbreeding between populations and accelerate their genetic differentiation (Via, 2009, 2012). It is possible for RI to evolve within a population, for example through polyploidy or chromosomal rearrangement, as long as the mutant individuals can reproduce by selfing or mating with other mutants, but have greatly reduced chance of successfully combining chromosomes with wildtype members of the population (Ptacek et al., 1994; Guelbeogo et al., 2005; Wood et al., 2009; Twyford and Friedman, 2015).

Following Dobzhansky’s pioneering experiments in the early 1900s that involved introgressing small regions of the genome from one species to another (Dobzhansky, 1936), “speciation genes” have been identified which confer low fitness in hybrid genetic backgrounds but not in their original genetic backgrounds. The genomic incompatibility caused by these genes stems from antagonistic interactions between parental genomes. Empirical studies on speciation genes have agreed on four general patterns (Coyne and Orr, 2004; Welch, 2004; Presgraves, 2010). First, the completion of RI can involve multiple incompatibilities. Second, the evolution of each incompatibility can be driven by multiple substitutions. Third, these substitutions are often found in uniparentally inherited genes. Fourth, these substitutions cause significant decrease in individual fitness only when they are in particular combinations.

Most of the models developed to account for these patterns are derivatives of the Dobzhansky–Muller-Incompatibility (DMI) model (Dobzhanksy, 1937; Muller, 1942). The basic argument of a DMI model is that combinations of alleles found in high frequencies in a given population must be compatible with each other; otherwise they would be removed from the population by purifying selection. Given that any individual unfortunate enough to inherit a deleterious combination of alleles has lower fitness, so will pass on fewer copies of those alleles to the next generation, we expect incompatible alleles to reduce in frequency within an interbreeding population. But alleles in one isolated population are not tested against alleles in other populations, so combinations of alleles that can only be formed by hybridization between populations may have low overall fitness. The DMI model is particularly good at explaining the four general patterns of “speciation genes,” because it predicts that incompatibility is a feature of particular allele combinations, and it assumes that each incompatibility involves multiple substitutions as it requires different substitutions in different populations (Welch, 2004). Note that, although the DMI model was originally proposed to describe the evolution of genomic incompatibility, it can be generalized to model the other two ways that substitutions can contribute to reproductive isolation through prezygotic incompatibility or local adaptation. We can use the DMI model in any cases by where substitutions have positive or neutral effects on fitness in their own populations, but hybrids may have deleterious combinations of alleles from both parent populations and so contribute to RI.

When a population is divided, each subpopulation will acquire substitutions, and every substitution has some chance of creating incompatibility between the subpopulations. Orr (1995) formulated the DMI model by assuming that each derived allele in a population has equal probability of being incompatible with each of the derived alleles in the other population. As a result, the number of incompatibilities is the number of all possible combinations of derived alleles, which increases with the square of the number of substitutions fixed between the two populations. This prediction that the rate of acquisition of RI increases quadratically with genetic differences between populations is called the snowball effect (Orr, 1995). While empirical studies have given some support for the snowball effect (Matute et al., 2010; Moyle and Nakazato, 2010; Wang et al., 2015), it has been suggested that these results suffer from inaccurate estimates of genetic divergence between species (Stadler et al., 2012) or overestimates in the frequency of hybrid inviability (Barbash, 2011).

The assumption that every derived allele in a population has equal probability of being incompatible with each of the derived alleles in the other population does not seem realistic, as we might expect alleles associated with the same function to have a greater risk of being incompatible than substitutions affecting distinct functions that do not interact. For example, if different copies of a duplicated gene are silenced by mutations in different populations, then a hybrid may inherit both silenced copies and so lose the gene function (Masly et al., 2006; Bikard et al., 2009). Similarly, if one population fixes two alleles in succession, one of which compensates for the deleterious effect of the other allele, then a hybrid may suffer reduction in fitness if it inherits only one of the alleles without the compensating effect of the other allele. Some speciation genes are associated with the suppression of molecular drive by cytoplasmic genomes, pathogens or selfish genetic elements, which favor their own transmission at the expense of fitness of gametes not carrying them (Johnson, 2010; Presgraves, 2010). A hybrid between two separate populations might have incompatible sets of alleles, having the elements from one population but the suppression mechanisms of another. Situations such as these that involve pairs of compatible alleles could drive a linear relationship between the number of incompatibilities and genetic divergence between populations. So if paired substitutions affecting related functions are the primary cause of genomic incompatibility, then we might expect the rate of increase in RI to be less than quadratic.

But we should not expect all incompatible alleles to be paired up. Indeed, observation suggests that multiple substitutions are often needed to confer an incompatibility (Coyne and Orr, 2004; Welch, 2004). Given the complexity in the way derived alleles interact to cause incompatibilities, we might not expect either a strict linear or quadratic increase in RI with the number of substitutions fixed between two populations. If incompatibilities can be identified from genetic mapping data, then the number of incompatibilities can be regressed against the number of substitutions fixed between species (Matute et al., 2010; Moyle and Nakazato, 2010). Then we could use the degree of greater-than-linear increase as a continuous measure of how many alleles in one population can be incompatible with each allele in another population. We can also explicitly model the accumulation of incompatibilities along phylogenies under different models and compare the goodness-of-fit between models against the observed number of incompatibilities among species (Wang et al., 2013).

We have seen that species differ in the rate of supply of new mutations, and that this should influence the rate of acquisition of substitutions. Isolation between populations – whether by physical, behavioral or genetic barriers to interbreeding – will cause different sets of substitutions to accumulate in sister populations. These substitutions must be compatible with other alleles in the population, but may be incompatible with substitutions accumulated independently in the sister populations. Now we will consider how the accumulation of incompatible substitutions leads to the formation of new species.

## Incompatibility Leads to Speciation

Both spatial isolation and genetic isolation may drive speciation if the isolating conditions can be sustained for a sufficiently long time. The DMI model suggests that isolation makes speciation inevitable, as divided populations will eventually accumulate sufficient differences to prevent the formation of successful hybrids during secondary contact. Even if RI is not complete during secondary contact, as long as there is some form of selection against hybridization and the selection is strong enough, traits that facilitate premating isolation can be selected for to complete RI (Servedio and Noor, 2003; Otto et al., 2008; Bank et al., 2012). The more substitutions contribute to RI, the stronger is the selection against hybridization. So in order to link mutation, substitution and speciation, we need to ask how the rate of accumulation of substitutions is linked to the rate of formation of reproductive isolation.

To answer this question, Gavrilets and Gravner (1997) extended the DMI model to the holey landscape model, which considers an adaptive landscape in which the “holes” are unfit genotypes. The holey landscape model demonstrates the conditions under which incompatible substitutions can be fixed in different populations, and provides quantitative predictions concerning the number of substitutions and the amount of time required to reach RI. Because strongly deleterious mutations are unlikely to be fixed, we need to look for the conditions under which incompatible substitutions can be fixed in different populations without incurring large fitness costs. In other words, we want to find evolutionary paths that move along the adaptive landscape without falling into a hole. Given a DMI prediction on the relationship between incompatibilities and substitutions, the holey landscape model is able to predict the number of evolutionary paths that a genotype can move along (Gavrilets, 2004).

When all the incompatible substitutions are paired, the number of incompatibilities is a linear function of the number of substitutions 𝜀d, where 𝜀 is the probability that each substitution causes reduction in fitness and *d* is the number of substitutions that differ from a starting genotype. If the starting genotype has *L* mutational targets, there are *L* number of steps the genotype can take with one substitution and the probability that any step will not lead to a hole is the chance that the next substitution is compatible with any previous substitutions and so causes no reduction in fitness: 1–𝜀. Then the expected number of paths for the starting genotype is *L*(1–𝜀). So the conditions where the starting genotype can move along the fitness landscape without falling into a hole is when *L*(1–𝜀) > 1, which is almost always true. Following similar arguments, the conditions for a snowball effect (quadratic increase in incompatibility) is 𝜀 < ln*L*/*L* (Gavrilets, 2004). In this case, having more mutational targets (*L*) should have lower values of 𝜀 to fulfill the condition of a snowball effect. In principle, 𝜀 can be estimated from a regression model between the number of incompatibilities and the number of substitutions fixed between species.

Assuming the simplest case where any one pair of incompatible substitutions causes complete RI, and all substitutions have the same chance of being incompatible, we can consider the probability that two populations differ in *d* substitutions can still interbreed. Two populations can interbreed if the path of *d* substitutions between them along the landscape does not fall in a hole, the probability of which is (1 - 𝜀)^d^. The probability that the *k*-th substitution causes RI is (1 - 𝜀)^k-1^𝜀. So the expectation of the number of substitutions required to complete RI is 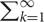 k(1 - 𝜀)^k-1^𝜀 = 1/𝜀. The expected amount of time to complete RI is then the ratio between the number of substitutions to reach RI and the substitution rate (Gavrilets, 2004). This model allows us to make predictions concerning the relationship between mutation rates, substitution rates and time to speciation. If each step along the evolutionary paths of the populations is neutral or nearly neutral, the overall substitution rate in a population should be primarily determined by the mutation rate (see Mutation Rate Influences Substitution Rate). If the incompatible substitutions are the result of adaptation, then this will reduce the time to reach complete RI, proportional to the product between effective population size and selection coefficient (Gavrilets, 2004). Because this calculation is based solely on new mutations and neglects the role of standing genetic variation within populations, it may overestimate time to achieve RI (Gavrilets, 2004). To account for standing genetic variation, one can numerically model changes in genetic variation both within and between populations (Gavrilets, 1999).

Clearly, the waiting time to speciation will depend on many interacting factors, including the rate of supply of genetic variation, the level of gene flow between isolated populations, the nature of the genetic changes underlying reproductive isolation, population size and nature of selective pressures. The holey landscape model only provides general predictions on the relationship between mutation rate, substitution rate, and speciation rate. But general predictions are useful for comparative studies, in which the influences of confounding factors may be treated as random effects when sample size is large.

Now that we have seen how species differ in mutation rates, and how these will influence the rate at which populations acquire substitutions that will cause them to become genetically incompatible, we can explore the relationship between the formation of genetically isolated populations and the rate of diversification. To do this, we need to consider the possible evolutionary fates of newly isolated populations once they have formed.

## Speciation Drives Diversification

Diversification is the net result of the processes that change the number of independent evolving lineages. The possible component processes of diversification are speciation adding lineages, extinction removing lineages, and merger of existing lineages through hybridization. So to understand how the processes that lead to genetically isolated populations contribute to diversification, we need to consider the factors that influence whether these new isolated populations will persist. If they persist, they may potentially divide again. Or, one or more of them may go extinct, resulting in the loss of any unique substitutions that had accumulated in that species. Or, if RI is not complete, isolated populations may reconnect and merge genetically with each other during secondary contact, losing their separate identity and becoming a single intermixed lineage.

While the process of speciation is typically studied by comparing closely related populations, diversification is usually studied by comparing the diversity of lineages over time and space. Because we are focusing only on evolutionary analysis of DNA sequences, we will not attempt to consider the ways that palaeontological, taxonomic and biogeographic data are used to shed light on the process of diversification. Instead we will consider only how diversification rate is measured from molecular phylogenies. Rather than considering the existence of RI between populations, phylogenetic studies of diversification rate typically rely on counting the number of recognized taxa within genetically distinct lineages. Speciation rate is typically estimated from the distribution of branching events in a phylogeny (e.g., Pagel et al., 2006), and differences in the net diversification rate estimated by comparing the number of extant species per lineage (e.g., Bromham et al., 2015).

These phylogenetic measures of diversification rate do not map exactly to the process being considered in speciation models. Taxonomic counts of species in a lineage are typically based on the number of physically or biogeographically distinct forms rather than direct measures of reproductive isolation. Phylogenetic identification of species recognizes populations that show deep genetic divergence (Simpson, 1962; de Queiroz, 1998), but not all the genetic differences between populations cause RI, and in some cases previously isolated populations can rejoin and fuse (Coyne and Orr, 2004), and physically or spatially distinct forms may be connected by interbreeding populations (Irwin et al., 2001). So phylogenetic estimates of diversification are not direct measures of time to reach RI as predicted by studies on speciation (Wiens, 2004).

To understand the relationship between phylogenetic patterns and speciation processes, we need to consider the interplay between population isolation, secondary contact, and reproductive isolation, and the relative amounts of evolutionary time between them. First we can define the expected waiting time between isolating events, which we will call *T_I_* (time to isolation). If isolating events are physical barriers preventing interbreeding, then *T_I_* may depend on chance environmental factors that divide populations. If isolating events are caused by substitutions that cause non-random mating, then *T_I_* depends not only on the occurrence of environmental factors that trigger divergent selection on the subpopulations, but also the amount of time the populations take to evolve non-random mating.

Similarly, we can consider the expected waiting time for two previously divided populations to come back into contact, reestablishing the potential for gene flow (*T_S_*, time to secondary contact). In the case of physical barriers, *T_S_* could depend, like *T_I_*, on chance environmental factors that remove isolating barriers (e.g., a river changing course), or *T_S_* may be due to ecological succession (e.g., burnt forest re-growing). Alternatively, *T_S_* might be governed by genetic change within the population, if one or both of the divided populations is able to adapt to the intervening unsuitable habitat. So when populations are isolated by unsuitable habitat, *T_S_* equals the length of time the intervening habitat remains unsuitable or the amount of time it takes for populations to adapt to the unsuitable habitat, whichever is shorter. When isolation is due to non-random mating, *T_S_* may be rapid (e.g., if a sudden change in environment removes divergent selection) or more gradual (e.g., if the breakdown in divergent selection is due to selection for the same new adaptive trait in both populations).

Finally, we can consider the waiting time to complete RI, *T_R_*, which describes the amount of time needed to accumulate sufficient incompatibilities to become irreversibly genetically incompatible, even if secondary contact between populations is restored. At *T_R_*, members of the population can no longer interbreed even if brought back into contact.

**FIGURE 2:**
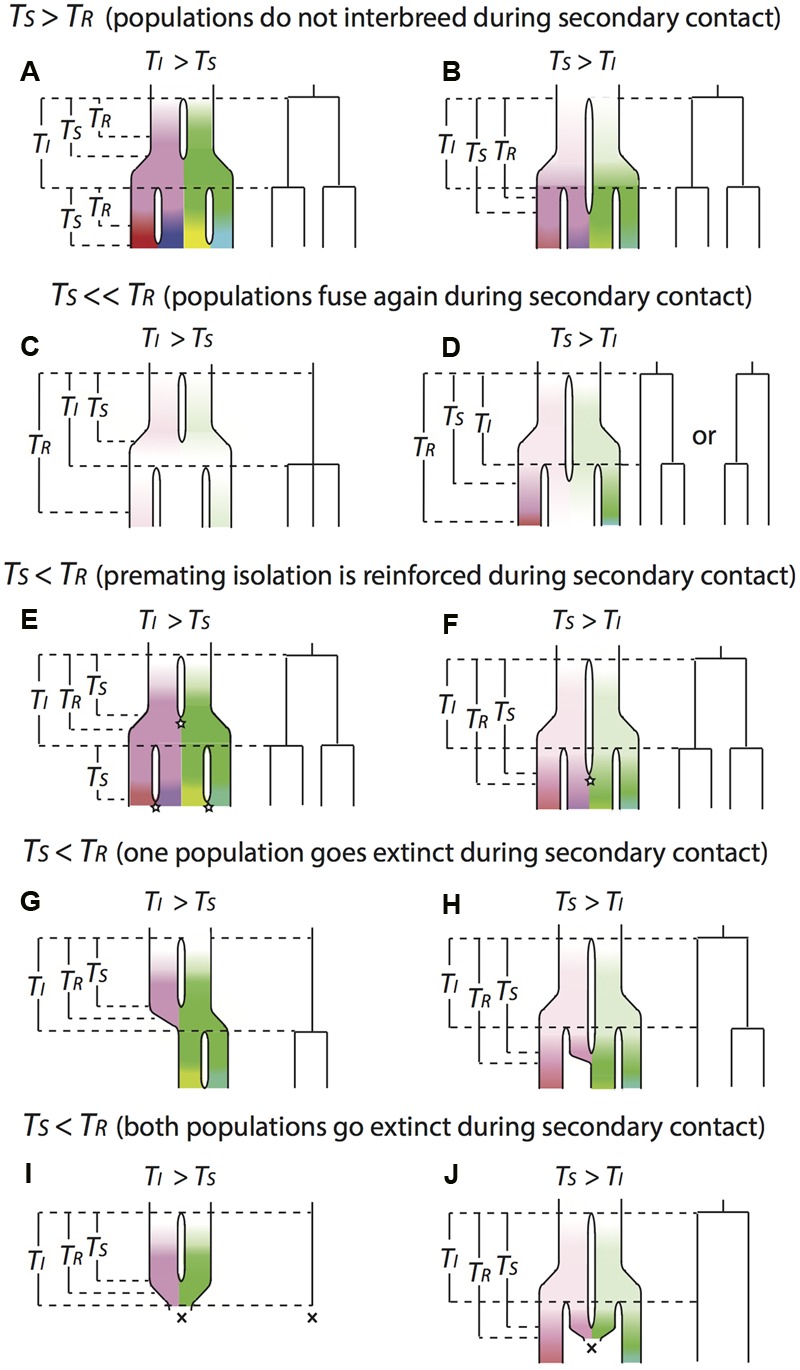
**Illustration for the conceptual approach to relating events of reproductive isolation to bifurcations on molecular phylogenies.** Each scenario includes the true history of diversification on the left and the reconstructed molecular phylogeny on the right, assuming the phylogeny is accurately reconstructed. Gaps between lineages in the true history indicate population isolation. The color indicates the genetic distinctiveness of each lineage (in terms of how many different substitutions the populations have acquired). The brighter the color is, the more distinctive the lineage is to the others. Diversification starts with a white lineage that bifurcates into a pink and a green lineage. During the process, populations are expanding and encounter new isolation events. Then pink lineage bifurcates into a red and a purple lineage, and green lineage bifurcates into a yellow and a blue lineage. *T_I_* is the expected waiting time between isolating events, which are kept the same under all the scenarios in order to make the internodal branch lengths of the reconstructed phylogenies under different scenarios comparable to each other. *T_S_* is the amount of time that populations remain isolated. *T_R_* is the amount of time that populations accumulate enough incompatibilities to reach complete RI. Stars represent reinforcement events and crosses represent extinction events. **(A–J)** describe different evolutionary scenarios: see text for details.

We can consider the pattern of lineage evolution under different relative values of *T_I_, T_S_* and *T_R_* (**Figure 2**). When the waiting time to secondary contact tends to be greater than the time needed to complete RI (T_S_ > T_R_), previously isolated populations won’t be able to interbreed if they come into secondary contact. In these circumstances, isolation leads to distinct species, and the internodal branch lengths of an accurately reconstructed phylogeny should equal T_I_ (**Figures 2A–B**). In this situation, the diversification rate estimated from branching events reflects the frequency of the isolating events.

When waiting time to secondary contact is much less than the time needed to develop complete reproductive isolation (T_S_ ≪ T_R_), previously isolated populations can interbreed if they come into secondary contact. If waiting time to secondary contact is shorter than the typical period between events that separate populations (T_I_ > T_S_), then separated populations are able to fuse again before the next isolation event happens. If we were to reconstruct this history on a phylogeny, we would not be able to detect the period of isolation, instead we would detect only the most recent isolating event that causes a split (**Figure 2C**). If waiting time to secondary contact is longer than the period between isolating events (T_I_ < T_S_), the next isolation event happens before populations have a chance to merge, keeping the most spatially distant populations isolated (**Figure 2D**). If we were to reconstruct this history on a phylogeny, we would observe the most spatially distant populations having deepest genetic divergence (even though they may not be the oldest isolating events), and the phylogenetic relationships between these populations and the population that is spatially distributed in the middle of them might not be fully resolved.

When waiting time to secondary contact is slightly less than the time needed to develop complete reproductive isolation (T_S_ < T_R_), some degree of reproductive isolation has evolved between populations, so hybrids produced by secondary contact will have reduced fitness, in which case there are two possible outcomes. First, traits that facilitate premating isolation may be selected for to prevent production of low-fitness hybrids, and this selection will favor mutations that bring about complete RI. Under this scenario, we would observe similar phylogenetic patterns as the case of T_S_ > T_R_ (**Figures 2E,F**). Second, the loss of reproductive output on unsuccessful hybrid mating could be severe enough to result in extinction of one or both populations (Todesco et al., 2016). If one population goes extinct, we would observe similar phylogenetic patterns as the case of T_S_ ≪ T_R_ (**Figures 2G,H**). If isolation events are less frequent than secondary contact events (T_I_ > T_S_; **Figure 2I**), then both populations may go extinct on merger due to loss of reproductive output. However, an isolation event may save a population from extinction by hybridization, allowing persistence of the lineage, leading to a pattern of species with discrete spatial distributions. In this case each internode on the phylogeny would represent multiple isolating and merging events (**Figure 2J**).

In our illustration, we assumed that the values of *T_I_, T_S_*, and *T_R_* were constant over the phylogeny, but it might be more realistic to model them as random variables. If the values can change over time and between lineages, then all the different scenarios illustrated in **Figure 2** may be found in a single reconstructed phylogeny reconstructed from the sequence data of a species group.

Using our conceptual approach to relating events of reproductive isolation to bifurcations on molecular phylogenies, we can make some predictions about the role of mutation rate in shaping average speciation rates. As outlined in the previous section, we expect mutation rate to have a role in determining the rate of formation of reproductive isolation between populations, so higher mutation rate should result in shorter *T_R_*. When the rate of secondary contact is controlled primarily by environmental change, populations with shorter *T_R_* are more likely to evolve reproductive isolation before secondary contact, so these populations will show the phylogenetic patterns under the scenario of T_S_ > T_R_ (**Figures 2A,B**), where internodal branch lengths are shorter and speciation rate is higher than other scenarios. Higher mutation rate may also result in faster evolution of non-random mating under divergent selection and so shorter *T_I_*. Populations with shorter *T_I_* are more likely to show the phylogenetic patterns under scenarios of T_1_ < T_S_ (**Figures 2B,D,F,H,J**), where speciation rate is also higher than the corresponding scenarios of T_1_ > T_S_ (**Figures 2A,C,E,G,I**). As a result, greater mutation rates may lead to faster speciation due to more rapid accumulation of reproductive isolation and/or faster evolution of non-random mating. But when secondary contact is driven by one or both of the divided populations evolving to occupy the intervening habitat, we would expect populations with faster mutation rate to have both shorter *T_S_* and *T_R_*, because greater genetic variation in populations may speed both adaptation to novel environments and the evolution of reproductive isolation. Under this situation, whether faster mutation can accelerate speciation depends on the relative magnitude of *T_S_* versus *T_S_*, which seems unlikely to have a general pattern.

It is also possible that mutation rate could influence diversification rate through relative extinction rates. Several theoretical studies have suggested that populations with faster mutation rate should adapt to novel environments faster (Lynch, 1996; Barton and Partridge, 2000; Stockwell et al., 2003; Orr and Unckless, 2008). This may provide some reduction in extinction rates if extinction is largely driven by failing to adapt to environmental changes. However, some population genetic models have predicted an opposite effect, where the accumulation of deleterious mutations in extremely small populations causes extinction by “mutational meltdown” (Lynch et al., 1995; Lande, 1998). Due to difficulties in measuring the extinction rate for most lineages, it is difficult to test these hypotheses directly. In terms of the effect on diversification rates measured from phylogenies, extinction influences phylogenetic branch lengths because in most cases we expect to have direct evidence for only a relatively small proportion of all of the extinct species. Missing data from the extinct lineages increases internodal branch lengths in the phylogeny and decreases estimates of diversification rate.

## Linking Mutation Rates to Diversification Rates

We have discussed how the rate of supply of new genetic variation is determined by the mutation rate that varies between species. We have also seen how patterns and rates of fixation of mutations in populations are influenced by factors that are in themselves shaped by species traits. Therefore we expect that patterns and rates of accumulation of genetic differences will differ between lineages. The acquisition of substitutions causes populations to diverge from each other, until they become so different that they cannot freely interbreed. These links – more mutations, more substitutions, more incompatibilities, a higher rate of speciation, and a higher rate of diversification – connect biochemical events in single cells to the generation of biodiversity. This is not to say that the production of variation by itself explains the process of diversification. Clearly, individual speciation events will be driven by particular local circumstances and the biology of the organism in question. But given that a link between mutation and diversification has been detected in comparative studies, we conclude that the production of variation makes some degree of contribution to the rate of evolutionary change at both the microevolutionary and macroevolutionary levels.

Empirical support for this connection between mutation and diversification comes primarily from molecular phylogenetic studies that show a correlation between estimates of rate of molecular evolution (estimated from phylogenies either from the tips, all branches, or root-to-tip paths) and measures of net diversification rate (either species richness or number of nodes in a tree). Association between rates of molecular evolution and species richness has been noted for a wide range of plants and animals (Barraclough and Savolainen, 2001; Davies et al., 2004; Pagel et al., 2006; Eo and DeWoody, 2010; Lancaster, 2010; Duchene and Bromham, 2013; Ezard et al., 2013; Bromham et al., 2015; Dugo-Cota et al., 2015). While individual studies may be subject to measurement biases or analytical artifacts, the diversity of approaches taken and the wide variety of data analyzed supports the contention that these studies reveal a widespread phenomenon.

While comparative studies demonstrate statistically significant and consistent patterns across many taxa, they don’t reveal the underlying cause of the relationship between rates of molecular evolution and diversification. We have hypothesized how mutation rate could promote diversification, either by providing more raw materials for adaptation, or by contributing to the evolution of reproductive isolation, or both. Conversely, it has been suggested that the process of speciation could cause acceleration in rates of molecular evolution, resulting in longer phylogenetic branch lengths in more species-rich lineages (Pagel et al., 2006). However, this hypothesis is difficult to reconcile with studies that have identified a correlation between the synonymous substitution rate and species richness (Barraclough and Savolainen, 2001; Lanfear et al., 2007; Duchene and Bromham, 2013; Bromham et al., 2015). Given that variation in synonymous substitution rate is considered to reflect differences in the mutation rate, it is difficult to see how speciation can directly influence mutation rate.

Alternatively, the association between molecular rates and diversification rate may be an incidental consequence of both being associated with some other factor, although it is difficult to think of a convincing indirect link. While body size and other associated life history characteristics correlate with rate of molecular evolution in a wide range of taxa, size is a surprisingly poor predictor of species richness in animals (Owens et al., 1999; Orme et al., 2002; Stuart-Fox and Owens, 2003; Isaac et al., 2005). Population size could offer an indirect link, if greater rates of speciation or extinction were associated with consistent reduction in population size, which could increase the fixation of slightly deleterious substitutions, however, there is currently little empirical evidence to support either a consistently lower population size or increased dN/dS in species-rich lineages (Bromham et al., 2015).

Various attempts have been made to provide a general theory of the tempo and mode of evolution by following the causal chain from biochemical processes to macroevolutionary change, for example by linking available kinetic energy to individual metabolic rate to both mutation rate and generation time, which may then influence the rate of evolutionary change (Gillooly and Allen, 2007). Such theories have been used to explain spatial patterns in biodiversity, on the assumption that higher temperatures drive greater rates of genetic change, either directly through an effect on rate of biochemical reactions, or indirectly through faster life histories reducing generation time (Rohde, 1992; Gillman and Wright, 2013). If rate of phenotypic evolution or niche change is increased by higher mutation rate or faster generation turnover, then lineages in warmer environments might diversify more rapidly (Smith and Beaulieu, 2009). However, theories linking energy availability to molecular change to diversity have been challenged on both empirical (noting exceptions to the rule) and theoretical grounds (questioning the validity of the underlying assumptions) (Duncan et al., 2007; Price et al., 2012). The proposed links in the causal chains might often be overwhelmed by other evolutionary forces operating on particular species (Dowle et al., 2013; Bromham et al., 2015; Glazier, 2015).

In particular, we might expect some of the evolutionary feedback loops discussed in this paper to have an impact on the knock-on effects of environmental temperature on rate of molecular evolution. Species are not passive in the face of environmental variation in mutagens such as UV or temperature, instead there is evidence that they adapt to their local conditions (Albarracin et al., 2012; Miner et al., 2015; Svetec et al., 2016), which may iron out some of the predicted environmental variation in mutation rates. Similarly, while variation in growth rates and generation turnover may influence the rate of accumulation of DNA replication errors, it seems that copy frequency effects are modulated by selection on copy fidelity mechanisms in order to produce acceptable levels of per generation error rates (Drake et al., 1998; Bromham, 2011; Sung et al., 2012). Life history and mutation rate must be matched to the environment and optimized to each other if a lineage is to persist through evolutionary time.

## Conclusion

The search for simple unifying theories in macroevolution and macroecology seems unlikely to succeed given the vast number of factors that can influence a particular lineage’s evolutionary trajectory, including rare events and the weight of history. Patterns in biodiversity are shaped by a great many factors, both intrinsic and extrinsic to organisms. Both evidence and theory suggests that one such factor is variation in the mutation rate between species. But the explanatory power of the observed relationship between molecular rates and biodiversity is relatively modest, so it does not provide anything like the predictive power that might be hoped for in a unifying theory. However, we feel that the evidence is growing that, in addition to the many and varied influences on the generation of diversity, the differential rate supply of variation through species-specific differences in mutation rate has some role to play in generating different rates of diversification.

Consideration of the forces shaping molecular evolution provides one piece of an intricate macroevolutionary puzzle. Molecular phylogenetic analysis has given us the ability to be able to consider both molecular processes and diversification rates simultaneously, giving us a new tool with which to explore the connections between the supply of variation and the production of biodiversity. We can’t help but think that Darwin would be pleased with these new views of the evolutionary process that molecular analyses afford us, as it offers the potential to demonstrate the links in the Darwinian chain that connects variation between individuals to divergence between populations to the generation of biodiversity:

“It cannot be asserted that organic beings in a state of nature are subject to no variation; it cannot be proved that the amount of variation in the course of long ages is a limited quantity; no clear distinction has been, or can be, drawn between species and well-marked varieties. It cannot be maintained that species when intercrossed are invariably sterile, and varieties invariably fertile; or that sterility is a special endowment and sign of creation.... But the chief cause of our natural unwillingness to admit that one species has given birth to other and distinct species, is that we are always slow in admitting any great change of which we do not see the intermediate steps.” (Darwin, 1859)

## Author Contributions

XH and LB designed the work and wrote the article. XH developed models and conducted analyses.

## Conflict of Interest Statement

The authors declare that the research was conducted in the absence of any commercial or financial relationships that could be construed as a potential conflict of interest.
